# Cardiovascular Issues in Tyrosine Kinase Inhibitors Treatments for Chronic Myeloid Leukemia: A Review

**DOI:** 10.3389/fphys.2021.675811

**Published:** 2021-07-05

**Authors:** Marco Santoro, Salvatrice Mancuso, Vincenzo Accurso, Daniela Di Lisi, Giuseppina Novo, Sergio Siragusa

**Affiliations:** ^1^Department of Surgical, Oncological and Stomatological Disciplines, University of Palermo, Palermo, Italy; ^2^Department of Health Promotion, Mother and Child Care, Internal Medicine and Medical Specialties, PROMISE, University of Palermo, Palermo, Italy; ^3^Hematology Unit, University Hospital “Paolo Giaccone”, Palermo, Italy; ^4^Department of Cardiology, University Hospital Policlinico “Paolo Giaccone”, Palermo, Italy

**Keywords:** cardiovascular events, chronic myelocytic leukemia, cardiovascular risk, cardio-oncology, tyrosine kinase inhibitions therapy

## Abstract

Chronic myeloid leukemia (CML) is a myeloproliferative neoplasm driven by a fusion gene, encoding for the chimeric protein BCR-ABL, with constitutive tyrosine kinase activity. The use of tyrosine kinase inhibitors (TKIs) has drastically improved survival, but there are significant concerns about cardiovascular toxicity. Cardiovascular risk can be lowered with appropriate baseline evaluation, accurate choice of TKI therapy, improvement of modifiable cardiovascular risk factors through lifestyle modifications, and prescription of drugs for primary or secondary prevention. Which examinations are necessary, and when do they have to be scheduled? How often should a TKI-treated patient undergo which cardiology test or exam? Is there an accurate way to estimate the risk that each TKI may determine a cardiovascular adverse event in a CML patient? In a few words, how can we optimize the cardiovascular risk management in CML patients before and during TKI treatment? The aim of this review is to describe cardiac and vascular toxicity of TKIs used for CML treatment according to the most recent literature and to identify unmet clinical needs in cardiovascular risk management and complications in these patients. Regarding the TKI-induced cardiovascular toxicity, the full mechanism is still unclear, but it is accepted that different factors may play different roles: endothelial damage and atherosclerosis, metabolic impairment, hypertensive effect, glomerular impairment, and mast-cell disruption. Preventive strategies are aimed at minimizing cardiovascular risk when CML is diagnosed. Cardio-oncology units in specialized hematology centers may afford a personalized and multidisciplinary approach to the patient, optimizing the balance between treatment of the neoplasm and management of cardiovascular risk.

## Introduction

Chronic myeloid leukemia (CML) is a myeloproliferative neoplasm driven by a fusion gene, encoding for the chimeric protein BCR-ABL, with constitutive tyrosine kinase (TK) activity. The use of tyrosine kinase inhibitors (TKIs), since the appearance of the first-generation inhibitor imatinib, in 2001, has dramatically changed the management of the disease, impacting prognosis to the point of obtaining survival data comparable to that of the general population (Faderl et al., 1999; Druker et al., 2001). Second- and third-generation TKIs (nilotinib, dasatinib, bosutinib, and ponatinib) have been demonstrated to be faster and even more effective than imatinib in reaching deep molecular responses (Jabbour et al., 2015; Vener et al., 2020).

These striking results obtained by second and third generation TKIs, however, usually come along with a heavier toxicity profile. Among the various adverse events known to be associated with TKI therapies, adverse cardiovascular events (CVAEs) are relatively often reported in clinical trials and in real-life drug surveillance programs (Li et al., 2015; Moslehi and Deininger, 2015; Douxfils et al., 2016; Moslehi, 2016). Moreover, clinical trials have proved the possibility of interrupting TKI therapies in defined cases to be safe and convenient in terms of cumulative toxicity, even in those patients that may need to restart TKIs after a temporary interruption (Mahon et al., 2010; Hochhaus et al., 2016; Ross et al., 2018).

Even though it is nowadays fully accepted that exposure to anti-CML TKIs confers an excess risk of CVAEs in the patient, data on underlying mechanisms are still unclear and (more important and impacting), it is still not well established an effective preventive and treatment strategy.

Which examinations are necessary, and when do they have to be scheduled? How often should a TKI-treated patient undergo which cardiology test or exam? Is there an accurate way to estimate the risk that each TKI may lead to a CVAE in a CML patient? In a few words, how can we optimize the cardiovascular risk management in CML patients before and during TKI treatment?

A lot of risk scores, based on the evaluation of multiple variables, were validated to estimate the risk of developing a CV events in the general healthy population. Of note, some of these scores were not targeted at evaluating important outcomes like heart failure, stroke, or symptomatic peripheral artery disease (Aghel et al., 2017).

The aim of this review is to describe cardiac and vascular toxicity of TKIs used for CML treatment according to the most recent literature and to identify unmet clinical needs in cardiovascular risk management and complications in these patients.

## Mechanisms of Cardiovascular Damage Through TK Inhibition

The most commonly reported, though still not completely understood, tumor–treatment-related cardiotoxicity is that caused by anthracyclines (ANT). The known mechanism of cardiac damage by ANT resides in the drug mechanism of action itself, which results in reactive oxygen species (ROS) and nitrogen species hyperproduction, leading to calcium and iron homeostasis disruption, nuclear damage, and myocyte dysfunction. On the other hand, the ANT-mediated blockade of II-*beta*-topoisomerase can cause mitochondria impairment in cardiomyocytes and thus elevate their ROS damage susceptibility. The clinical presentation is a kind of acquired cardiomyopathy, referred to as “ANT-induced cardiomyopathy.”

Tyrosine kinase inhibitors-induced cardiovascular toxicity seems to have other roots, and even if the full mechanism of cardiovascular damage is still unclear, it is widely accepted that various factors may play different roles:

• *Endothelial damage and atherosclerosis*: nilotinib was demonstrated to inhibit human vascular endothelial and microvascular endothelial cell proliferation and survival, both in presence and absence of VEGF, and determining an increase of caspase-3 and -7 (markers of apoptosis), determining damage that partially resumes the one known to be caused by VEGF-inhibiting TKIs (Galvano et al., 2019). Moreover, nilotinib causes an increased expression of ICAM-1, VCAM-1, and E-selectin as pro-atherogenic surface molecules in endothelial cells in *in vitro* models. Imatinib was instead not capable of determining these endothelial alterations in the same study. A similar mechanism of damage may be inductively postulated for ponatinib, a potent VEGFR inhibitor. Human umbilical venous endothelial cells were tested *in vitro* with the five available TKIs, demonstrating how dasatinib and ponatinib induced apoptosis and necrosis also at lower concentrations than those used for the treatment of CML, while imatinib, nilotinib, and bosutinib did not, even after 72 h of exposure to the drugs (Hadzijusufovic et al., 2017; Haguet et al., 2020).
• *Metabolic impairment*: data from trials evidence that second-generation TKI nilotinib often determines glucose level elevations and sometimes triggers or worsens a preexisting type 2 diabetes mellitus, through the blocking of a post-insulin receptor pathway. In fact, ABL seems to be involved in the insulin pathway signaling, and its blocking by TKI may cause decreased insulin sensitivity and the need for an excess of insulin secretion. Moreover, exposition to nilotinib appears to decrease also adiponectin concentrations, thus further impairing tissues insulin sensitivity (Frasca et al., 2007; Genua et al., 2009).
• *Hypertensive effect*: Arterial hypertension in a common adverse event during treatment with nilotinib and ponatinib. Both of these drugs potently inhibit VEGFR among the other TK. VEGF physiologically supports nitric oxide (NO)-dependent arterial vasodilation and upregulates NO production acting on the endothelial cells, thus regulating the basal arterial tone and pressure levels. VEGF and VEGFR inhibitors determine the blocking of this mechanism and may cause hypertension (Ku et al., 1993; Hood et al., 1998). Arterial hypertension may itself act as a potential risk factor for other and more severe CV events, such as myocardial infarction and stroke.
• *Glomerular impairment*: VEGF also plays a role in the kidneys in cellular proliferation and homeostasis. The inhibition of its pathway may lead to glomerular dysfunction, determining or worsening preexisting proteinuria and hypertension (Kappers et al., 2009; Lankhorst et al., 2015; Li et al., 2015).
• *Mast-cell disruption*: Mast cells have an important role in vascular tissue repair, producing and releasing heparin and other bioactive tissue plasminogen activators. Both nilotinib and ponatinib have KIT-inhibiting activity and thus may impair mast cell function. Imatinib has a KIT-inhibiting activity as well, but it has been demonstrated to be unable to impair endothelial cell regeneration and to sensitize the endothelium to atherosclerosis generation, suggesting that mast cell impairment alone is not sufficient to determine a pro-atherogenic phenotype in endothelial cells (Genua et al., 2009).

Indeed, the mechanisms involved are due either to the inhibition of the main target of the molecules used to cure CML (ABL inhibition, “on-target” effects) and of other kinases that are not involved in the disease’s pathogenesis (“off-target” effects; Force and Kolaja, 2011). However, it is a common idea that off-target effects are more involved in the determination of cardiovascular risk increase, in that first-generation TKI imatinib appears even to be protective, improving the metabolic profile of patients with diabetes and not affecting the age-adjusted cardiovascular risk profile (Moslehi and Deininger, 2015).

Moreover, every TKI may have a different spectrum of cardiac and/or vascular toxicity in different patients, according to their age, sex, comorbidities, and the presence of additional conventional and non-conventional cardiovascular risk factors (i.e., smoking habits, dyslipidemia, overweight, familiarity, diabetes, assumption of other therapies that may have CV toxicity as known adverse events, and so on).

The conventional risk factors for CVAEs are traditionally divided into modifiable and non-modifiable factors: respectively, the first group includes dyslipidemia, hypertension, cigarette smoking, obesity, and physical inactivity; the second group includes age, sex, ethnicity, and family history. These risk factors have been extensively proved to be significant in a lot of population-based trials and on large-scale case–control studies (Yusuf et al., 2004; D’Agostino et al., 2008). Among the so-called non-conventional CV risk factors, high-sensitivity C-reactive protein, interleukin-1 (IL-1), interleukin-6 (IL-6), fibrinogen- and lipoprotein-associated phospholipase A2, homocysteine, and lipoprotein (a) have been studied, and elevation of the corresponding values appears to have a statistical association with the occurrence of CVAEs in the general population (Emerging Risk Factors Collaboration Kaptoge et al., 2012).

The fact that CVAEs occur in CML patients while in TKI therapy with preexisting (or upcoming) non-TKI-related cardiovascular risk corroborates the hypothesis that primary prevention is the key strategy in reducing the risk of developing a CVAE for these patients. The most recent 2020 ELN recommendations for CML diagnosis and treatment report cardiovascular adverse events as the main non-hematologic AEs and strongly recommend avoiding the choice of ponatinib and nilotinib in patients with previous or concomitant arterial vascular disease, even if the authors themselves add the phrase “unless there is unique need” to the recommendation (Hochhaus et al., 2020).

In 2016, Dorer et al. demonstrated how, after adjusting covariates, there was a significant association between the occurrence of cardiac failure and CVAEs and ponatinib dose intensity in the PACE trial. In particular, the odds ratio (OR) for cardiac failure was more than 2, for arterial thrombotic events was more than 1.5, while for hypertension the OR was 1.3, weaker but still significant. The strongest independent predictors of arterial occlusive events were dose intensity, the history of an ischemic disease, and advanced age (Dorer et al., 2016).

## Available TKIs and Relative CV Risk Profile

In Table 1, the five available ABL-inhibiting TKIs were reports along with the known cardiovascular toxicity profile and mechanisms (see Table 1).

**TABLE 1:** Currently available ABL-inhibiting TKIs, their known cardiovascular-associated toxicity, and the possible underlying mechanisms.

TKI gen	Drug	Cardiovascular toxicity	Involved mechanisms
1st	Imatinib	Potential benefit on metabolic and cardiovascular function	NA
2nd	Nilotinib	Arterial hypertension, ischemic events	Increased P-selectin, sICAM-1, sVCAM-1, TNF-alpha, IL-6, PAR-1-mediated alpha granule release, endogenous thrombin levels (*in vitro* and *in vivo*), inhibition of endothelial cell proliferation, increased glucose levels, glomerular impairment, mast cell disruption (impaired tissue repair)
2nd	Dasatinib	Pulmonary arterial hypertension	Hypoxic pulmonary vasoconstriction responses reduction (possible)
2nd	Bosutinib	Potentially none	NA
3rd	Ponatinib	Peripheral arterial obstructive disease, other ischemic events	Not known. Hypothetically similar to nilotinib. Dose dependent.

### Imatinib

The first generation TKI imatinib is surely the most convenient BCRABL-inhibiting drug in terms of cardiovascular and metabolic toxicity. Imatinib therapy appears to be protective against insulin resistance and hyperglycemia (Agostino et al., 2011). In a large cohort of CML patients treated with imatinib versus initial placebo, the imatinib group had lower rates of peripheral arterial events (Giles et al., 2013). Preclinical data suggest that imatinib may lower and reverse pulmonary hypertension, so that the drug has been evaluated in several trials for pulmonary hypertension treatment (Ghofrani et al., 2005).

### Dasatinib

Vascular events during treatment with dasatinib typically affect the pulmonary vessels, often determining a clinical presentation of pulmonary arterial hypertension (PAH). Due to the complex diagnostic means by which PAH is diagnosed, data from the most important dasatinib trials do not include PAH evaluation and measurement. Dasatinib-induced PAH mechanisms still remain unclear, but there are some hints that chronic exposure to dasatinib may attenuate hypoxic pulmonary vasoconstriction responses and thus increase susceptibility to PAH. Despite this initial thinking, it seems that this mechanism is independent of Src-inhibition (Guignabert et al., 2016; Özgür Yurttaş and Eşkazan, 2018).

In 2012, the French Pulmonary Hypertension Registry reported nine patients with PAH related to dasatinib. From the result of the Registry, it was estimated that PAH happens in at least 0.45% of individuals who are chronically exposed to dasatinib. In those cases, the withdrawal from the drug determined significant improvements of the subjective and objective presentation (Montani et al., 2012).

Pleural effusion, which is not actually a cardiac or vascular toxicity, is, however, an adverse event that comes into differential diagnosis with cardiac impairment. The drug-related pathological mechanism is known to be an off-target Src kinase inhibitor, with the consequence of abnormal pleural fluid retention (Cortes et al., 2017). Another possible mechanism involved in pleural effusion determination during dasatinib assumption is dasatinib-triggered autoimmunity (de Lavallade et al., 2008).

### Nilotinib

Nilotinib exerts a much more potent imatinib-like ABL inhibition, thus resulting in better molecular responses with the possible cost of a worse safety profile. In terms of CV toxicity, nilotinib triggers a prothrombotic state, increasing thrombosis mediators such as P-selectin and potentiating the PAR-1-mediated alpha granule release. Furthermore, exams performed on CML patients’ peripheral blood revealed increased *ex vivo* platelet adhesive function, increased soluble P-selectin in these patients’ plasma, and increased levels of sICAM-1, sVCAM-1, TNF-alpha, IL-6 as well, along with the increase in endogenous thrombin potential levels *in vivo*, despite being on daily low-dose aspirin (Alhawiti et al., 2016; Bocchia et al., 2016; Hadzijusufovic et al., 2017).

### Bosutinib

The bosutinib cardiac and vascular risk profile is probably comparable to that of Imatinib. Despite the already low CV risk added by this TKI, it has to be highlighted that data on bosutinib cardiac and vascular toxicity contain the bias that the trials leading to its approval were performed on at least third-line CML patients, often heavily pretreated with other TKIs with the over mentioned CV toxicity. Moreover, bosutinib real-life data come from at least second-line CML patients and in second-line patients frequently in presence of a cardiovascular disease or of a moderate-to-high cardiovascular risk, which is commonly the reason that they are not suitable to second-line dasatinib, nilotinib, or ponatinib (Gambacorti-Passerini et al., 2014; Brümmendorf et al., 2016; Cortes et al., 2016).

### Ponatinib

Due to its high affinity to the ABL ATP-binding site, ponatinib is probably the most potent BCR-ABL-inhibiting drug in the treatment of CML. The pivotal PACE trial initially reported a high rate of cardiovascular severe adverse events, and the drug was temporarily suspended from commerce by the United States FDA and reintroduced when analysis of CV risk factors in patients who developed CV complications was completed and it was demonstrated that those occurred mostly in patients with pre-ponatinib high CV risk (Valent et al., 2017).

The 5-year follow-up report of the pivotal phase 2 Ponatinib Ph1 ALL and CML Evaluation (PACE) trial revealed cumulative rates of treatment-emergent arterial obstructive events of 31% (84 events) in the chronic phase CML (CP-CML) subgroup. Exposure adjusted events accounted for 14.1 events per 100 patients per year. In the evaluation of these data, we have to take trial-enrolled patients with CP-CML with intolerance or resistance to dasatinib or nilotinib, and the derived cohort of 270 CP-CML patients took ponatinib as at least the third line of TKI therapy in 57% of the cases. It is strongly accepted nowadays that CV toxicity of ponatinib is dose-dependent. It is notable that treatment-emergent arterial obstructive events occurred with lower rates in the reduced dosage group; in particular, the ponatinib dose at initial onset of the event was 45 mg in 42% of patients, 30 mg in 24%, and 15 mg in 26%. Initial onset of the first event occurred in the post-study follow-up in 7% of cases.

Moreover, risk factors for the development of serious arterial obstructive events were identified in the study: traditional CV risk factors, such as hypertension, hypercholesterolemia, diabetes, obesity, and a history of ischemic and non-ischemic cardiac disease. The gradual increase of the number of conventional and non-conventional risk factors determines the increment of the rate of arterial thrombotic events in the lower-level analysis, increasing from 0 to 1 and from 1 to 2 or more risk factors (Cortes et al., 2018).

In a recent report we published, the series of five patients with CML who harbored high global cardiovascular risk or had already experienced heart disease were reported to develop no CV adverse events after a median follow-up time of 34 months, assuming ponatinib at the lowest effective dose, when other therapies were not possible or available (Santoro et al., 2019).

## Preventive Strategies

The first step on the path to achieving an efficient cardiovascular prevention strategy is the correct patient evaluation and risk classification. The very important and sometimes problematic choice of one TKI instead of another has to be guided by disease features and patient characteristics as well, and it has to comply with the established purposes of therapy that come from that first assessment.

Pre-TKI cardiovascular risk assessment is probably one of the turning points and an important unmet clinical need in the field of CML cardio-oncology. One issue of significant relevance is which risk score is better to obtain affordable baseline CML patient evaluation in order to eventually exclude first-line nilotinib or ponatinib. There is no evidence that high CV-risk patients have to avoid dasatinib, even if the risk of PAH is known and present.

The extensively used SCORE charts have the pros of being targeted and standardized on populations from different areas, not limited to United States data, but they were validated to estimate 10-year risk of fatal events only and thus may be inappropriate for evaluating the global CV risk (Breccia et al., 2015). On the other hand, the Framingham risk score predicts the risk of developing a cardiovascular disease during the following 10 years but does not take into account PAOD, a commonly reported nilotinib and ponatinib vascular adverse event, as a possible outcome (D’Agostino et al., 2008).

Targeting the modifiable CV risk factors after CML diagnosis is undoubtedly the preferred strategy, despite the choice upfront of TKI.

Although low-dose aspirin prevents recurrent CV events in patients with previous diagnosis of CV disease, its role in primary prevention in patients with no CVD is still under consideration. Meta-analyses have revealed that primary prevention with low-dose aminosalicylic acid guarantees a 10–13% relative risk reduction in serious CV events, at the cost of a risk of intracranial and gastrointestinal hemorrhage (Capodanno and Angiolillo, 2016).

Even if relative contraindications are suggested by international recommendations for the management of CML, absolute contraindications based on cardiovascular risk are hard to accept for patients with multiple types of intolerance or resistance due to point mutations that require ponatinib as the only possible treatment. Real-life data support the use of reduced doses of second and third generation TKIs to maintain optimal responses in patients initially treated with a full dose, with the aim of minimizing dose-dependent cardiovascular toxicity.

Upfront dose reduction is still a taboo theme, even if pharmacokinetic analysis has indicated that ponatinib dose reduction to 15 mg a day upfront in CP-CML may confer advantages in terms of safety, while maintaining its efficacy (Molica et al., 2019).

Cardio-oncology units are available nowadays to refine the cardiological, basal, and follow-up evaluation of patients with hematological neoplasms, eligible for TKI therapies. Basal evaluation is preferable in all CML patients, despite the chosen first-line TKI. A cardiovascular risk stratification and multidisciplinary approach are essential steps to minimize the global CV risk for the patient and therefore optimize the possibility of fully treating the disease. Cardio-oncology follow-up schedules are still not standardized.

## Conclusion and Perspectives

Adverse CVAEs are still a major issue in CML treatment, becoming possible comorbidities in the landscape of an expected very favorable long-term outcome.

The availability of an appropriate CV risk score for baseline evaluation of CML patients eligible for TKI treatment is an unmet clinical need. The validation of a score that estimates the 10-year probability of developing fatal and non-fatal cardiovascular disease, including myocardial ischemia, cerebrovascular ischemia, peripheral artery obstructive disease, and cardiac death, is strongly desired.

Cardio-oncology unit referral at diagnosis and during the recommended follow-up is considered useful and advantageous in the global management of patients on nilotinib or dasatinib but is suggested also in CML patients assuming other TKIs.

Optimizing modifiable CV risk factors through lifestyle modification and eventual prescription of primary prophylactic drugs is the preferred strategy to be applied upon CML diagnosis, despite the TKI chosen. The use of low-dose aspirin for primary prevention may reduce the pre-TKI CV risk in selected patients.

## Author Contributions

MS and SM wrote the text and reviewed the literature. VA and DD collected the literature and reviewed the text. GN and SS equally supervised the project. All authors contributed to the article and approved the submitted version.

## Conflict of Interest

The authors declare that the research was conducted in the absence of any commercial or financial relationships that could be construed as a potential conflict of interest.
